# A Case Report of Mechanical Intestinal Obstruction Caused by Delayed Heterotopic Ossification of Postoperative Incision

**DOI:** 10.1002/ccr3.71947

**Published:** 2026-03-17

**Authors:** Ke Xie, Hengwei Cui, Ling Li, Honghu Xie, Jin Feng

**Affiliations:** ^1^ Department of Gastrointestinal Surgery The Third Affiliated Hospital of Soochow University Changzhou China

**Keywords:** abdominal incisional heterotopic ossification, abdominal surgical complication, intestinal obstruction, pathological calcification

## Abstract

Heterotopic ossification (HO) refers to the abnormal formation of mature lamellar bone within soft tissues where bone is not typically present, such as muscles, tendons, ligaments, and joint capsules. While commonly associated with trauma, spinal cord injury, or orthopedic procedures, abdominal incisional heterotopic ossification (AIHO) is exceedingly rare. Its pathogenesis may be closely related to local trauma, inflammatory responses, and genetic predisposition. AIHO typically occurs between 3 months and 5 years postoperatively, with an estimated incidence of 0.3%–1.2% among all HO cases. Ossified masses can compress or mechanically tether the intestines, resulting in bowel obstruction. Herein, we report a rare case of AIHO causing mechanical intestinal obstruction 20 years after a hysterectomy. The diagnosis was confirmed by abdominal computed tomography, and the patient underwent successful surgical resection. We also review the literature and summarize strategies for the management and prevention of this complication.

## Key Clinical Message

Abdominal incisional heterotopic ossification (AIHO) can cause mechanical bowel obstruction decades after surgery. Diagnosis requires computed tomography with bone‐window/3D reconstruction and histology. Complete surgical resection is curative. Postoperative NSAIDs reduce recurrence. High‐risk patients need NSAID prophylaxis.

## 1 Introduction

Heterotopic ossification (HO) is defined as the aberrant formation of mature lamellar bone in non‐skeletal soft tissues. While it is a well‐recognized complication, its incidence and clinical significance vary dramatically depending on the anatomical site and the inciting event. The most commonly occurs around joints, especially after trauma, spinal cord injury, or orthopedic surgery [1, 2]. In stark contrast, HO occurring specifically at abdominal surgical incision sites—termed abdominal incisional heterotopic ossification (AIHO)—is a considerably rarer entity.

The precise overall incidence of AIHO remains difficult to ascertain due to the scarcity of large‐scale epidemiological studies and a historical predominance of case reports in the literature. However, emerging evidence suggests its prevalence may be underestimated, particularly in certain high‐risk cohorts. For instance, a notable retrospective analysis of 152 patients undergoing abdominal surgery found radiographic evidence of HO in 25.7% of cases [3], indicating that AIHO is not an exceedingly rare phenomenon in this context.

The clinical relevance of AIHO lies not only in its potential to cause morbidity but also in the diagnostic challenge it presents. Many cases are asymptomatic and discovered incidentally. However, when symptomatic, AIHO can lead to chronic pain, a palpable mass, or, as in the index case, mechanical bowel obstruction due to adhesion, traction, or direct compression. This complication can manifest months to decades after the initial surgery, creating a diagnostic gap where late‐onset symptoms are not readily attributed to the prior operation. Consequently, AIHO can be misdiagnosed as more common conditions like adhesive ileus, potentially delaying appropriate management.

Given its potential to cause significant morbidity, its underappreciated frequency in high‐risk surgical patients, and the diagnostic pitfalls associated with its often‐delayed presentation, AIHO warrants greater clinical awareness.

In this report, we present a case of AIHO that led to mechanical intestinal obstruction two decades after a hysterectomy. We also provide an overview of potential management strategies based on a literature review.

## 2 Case History/Examination

A 75‐year‐old woman presented with acute periumbilical cramping pain, nausea, vomiting, and cessation of flatus and bowel movements for 2 days following the ingestion of sweet potatoes. Her medical history included hypertension and cerebral infarction. She had undergone a total hysterectomy for uterine fibroids 20 years prior. Physical examination revealed periumbilical tenderness and hyperactive bowel sounds with high‐pitched, metallic timbre—findings consistent with mechanical bowel obstruction.

**FIGURE 1:**
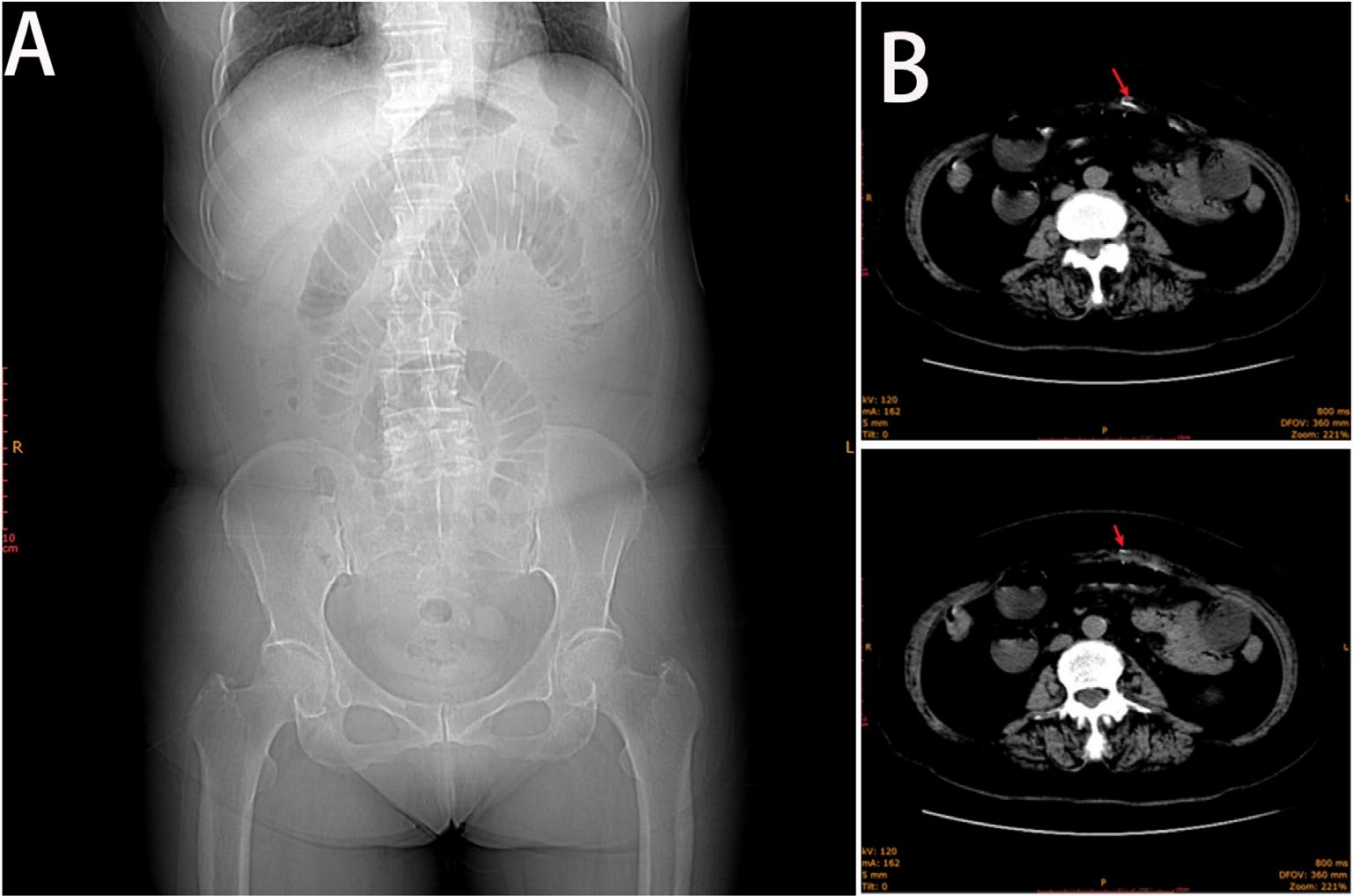
(A) CT localization image. (B) CT scan showing segmental dilation of the small intestine with intraluminal fluid and gas accumulation, formation of air‐fluid levels, and patchy high‐density shadow in the mid‐abdominal wall.

Laboratory tests showed metabolic acidosis (CO_2_CP: 19.2 mmol/L) and elevated urea (7.30 mmol/L). Abdominal computed tomography (CT) revealed dilated small bowel loops with air‐fluid levels and a patchy dense shadow in the mid‐abdominal wall (Figure 1A,B).

## 3 Differential Diagnosis

In the preoperative evaluation of this elderly patient with multiple abdominal surgeries and classic mechanical small bowel obstruction symptoms, adhesive ileus was initially the most probable diagnosis—consistent with the surgical principle that prior abdominal operations are the main risk factor for adhesive obstruction. Emergency CT findings of dilated small bowel loops with air‐fluid levels were in line with this common etiology. However, an atypical finding—a well‐defined “patchy dense shadow in the middle abdominal wall” (at the previous hysterectomy incision site)—prompted reevaluation of a sole diagnosis of simple adhesive disease. Unlike benign fibrous adhesions (typically soft tissue density), this focal dense shadow suggested alternative pathologies such as dystrophic calcification, calcified desmoid tumor, or HO.

Preoperative differentiation focused on the density's attributes. Even without bone‐window imaging or 3D reconstruction, the discrete, potentially calcified lesion in the abdominal wall scar differed from adhesive bands (usually invisible or non‐calcified linear strands). Thus, while adhesive obstruction remained the primary working diagnosis due to its high prevalence, the atypical lesion raised HO as a key differential, indicating the obstruction might be caused by a rigid, space‐occupying ossified mass (rather than simple fibrous bands) leading to bowel kinking or constriction.

Definitive exclusion of pure adhesive disease and confirmation of HO required surgical exploration and histopathology. The preoperative plan therefore included not only adhesion lysis for presumed adhesive ileus but also targeted exploration of the dense abdominal wall shadow, with preparation for resection if a solid mass was found.

Subsequent surgical findings—a hard, bony mass at the adhesion site—confirmed the preoperative suspicion from CT, effectively ruling out simple adhesive disease as the sole cause. The pathological report of an ossified nodule provided final conclusive evidence.

## 4 Conclusion and Results

**FIGURE 2:**
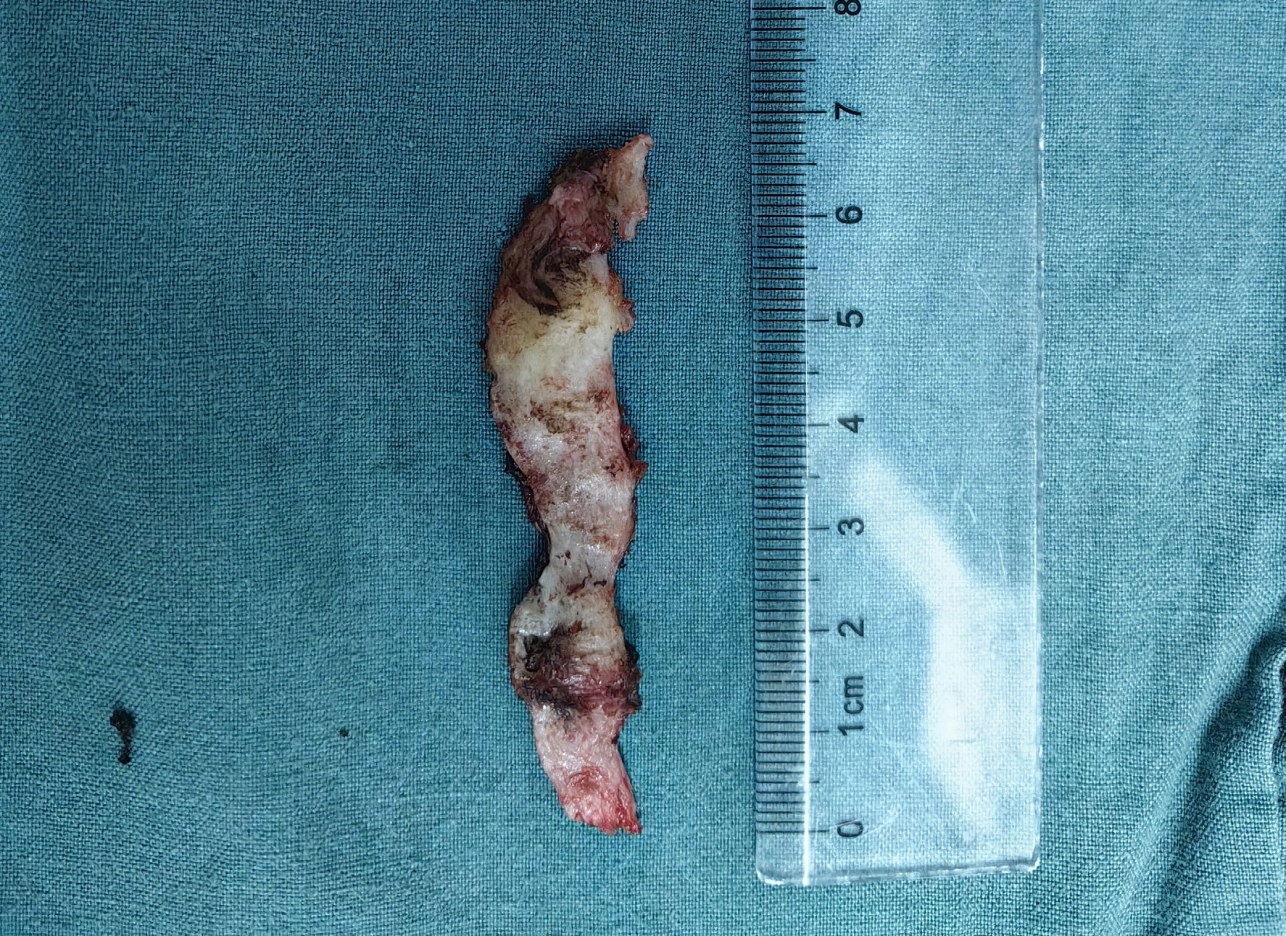
Intraoperatively photographed specimen images.

**FIGURE 3:**
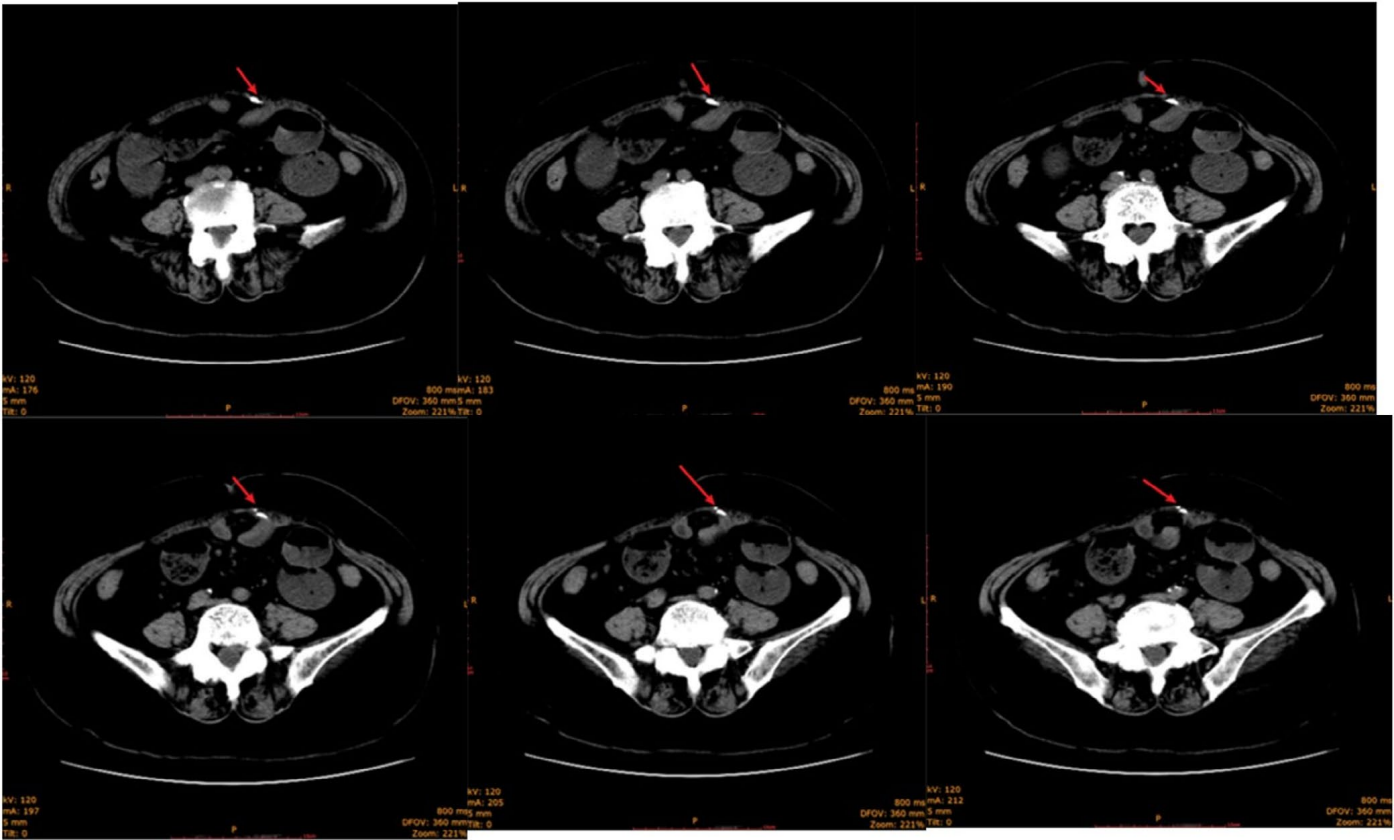
CT image showing an irregular mass embedded between the peritoneum and the posterior sheath of the rectus abdominis at the site of adhesion. The mass is well demarcated from the surrounding tissues but exhibits mild adhesion to the mesenteric vessels, resulting in angulation of the intestinal loop and proximal small bowel dilation.

The patient underwent emergency surgery. Surgical exploration via a midline lower abdominal incision revealed extensive adhesions between the ileum (approximately 200 cm from the ligament of Treitz) and the peritoneum beneath the prior incision. A hard, irregular mass (Figure 2) was embedded between the peritoneum and the posterior rectus sheath. The mass was grayish‐white, bone‐hard, and well demarcated from surrounding tissues, though mildly adherent to the mesenteric vessels. This caused angular torsion of the intestine and proximal bowel dilatation (Figure 3). The mass was excised in its entirety, followed by adhesiolysis and enterotomy decompression (800 mL of yellow‐green content). The abdominal cavity was irrigated with normal saline, and a pelvic silicone drain was placed prior to closure.

**FIGURE 4:**
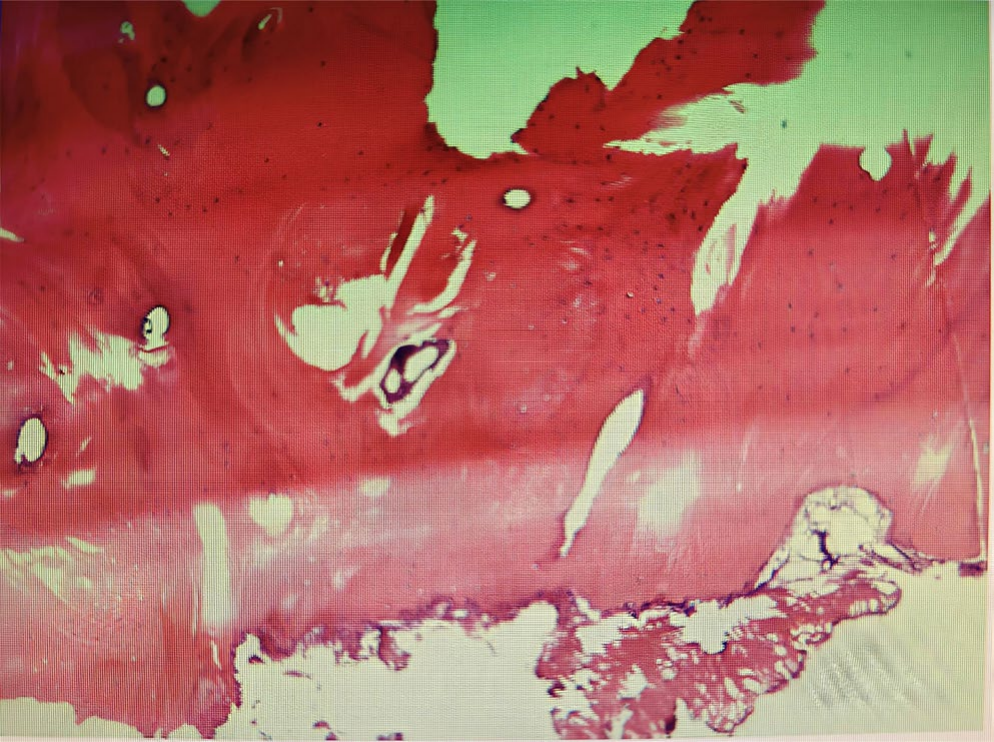
Postoperative histopathology (preperitoneal tissue) reveals an ossified nodule with intertrabecular hematopoiesis.

Postoperative histopathology showed a preperitoneal ossified nodule with intertrabecular hematopoiesis (Figure 4), consistent with HO. The patient resumed flatus on postoperative day 2 and bowel movements on day 4, followed by gradual reintroduction of diet. At the 1‐year follow‐up, no recurrence was detected on CT.

## 5 Discussion

This case identifies AIHO as a delayed cause of mechanical intestinal obstruction (20 years post‐abdominal surgery). AIHO is extremely rare (0.3%–2% overall incidence in abdominal surgery patients; 5%–12% in high‐risk subgroups: multiple abdominal surgeries, incision infection, metabolic disorders like diabetes/obesity), so universal screening/prevention is unnecessary (avoids overmedicalization), with a focus on targeted high‐risk identification [4].

AIHO diagnosis faces two challenges: nonspecific symptoms mimicking adhesive bowel obstruction and early calcifications easily misinterpreted on CT (3D reconstruction often needed; histological confirmation of bony trabeculae is definitive) [5, 6].

Surgical resection (complete ossified mass excision + meticulous adhesiolysis) is the main treatment; postoperative NSAIDs (e.g., celecoxib) reduce recurrence from 15%–20% to ~5% [7].

Optimized “stratified, resource‐adaptive” prevention for high‐risk patients:

1. NSAIDs (generic alternatives for resource‐limited areas) for 4–12 weeks post‐op (no routine use for low‐risk patients);
2. Imaging follow‐up: CT + 3D reconstruction (resource‐rich) or ultrasound/plain CT (resource‐limited, focusing on incision area);
3. Serum bone metabolism markers (auxiliary only for resource‐rich centers);
4. Laparoscopy (universal) + low‐cost anti‐adhesion materials for high‐risk patients.

In conclusion, AIHO should be considered in delayed mechanical bowel obstruction (especially high‐risk subgroups). Targeted interventions (not universal strategies) are meaningful. CT identification of lamellar bone + pathology confirms diagnosis; surgery + NSAIDs yield best outcomes, with optimized strategies feasible globally (including developing countries).

## Author Contributions

**Ke Xie:** conceptualization, data curation, project administration, validation, visualization, writing – original draft. **Hengwei Cui:** conceptualization, data curation, project administration, resources, supervision, visualization, writing – original draft. **Ling Li:** investigation, methodology. **Honghu Xie:** resources, validation, writing – original draft. **Jin Feng:** resources, validation, writing – original draft, writing – review and editing.

## Funding

The authors have nothing to report.

## Ethics Statement

The study was conducted in accordance with the Declaration of Helsinki, and approved by the ethics committee of the Third Affiliated Hospital of Soochow University (protocol code: CYLL220921).

## Consent

Written informed consent was obtained from the patient to publish this report, following the journal's patient consent policy.

## Conflicts of Interest

The authors declare no conflicts of interest.

## Data Availability

The data that support the findings of this study are available from the corresponding author upon reasonable request.
